# Spontaneous Pneumothorax in a Term Neonate

**DOI:** 10.7759/cureus.63999

**Published:** 2024-07-06

**Authors:** Badar Al Dhouyani, Amal R Al-Naimi

**Affiliations:** 1 Pulmonology, Sidra Hospital, Doha, QAT; 2 Pediatric Pulmonology, Sidra Medicine, Doha, QAT

**Keywords:** air leak syndrome, pneumomediastina, term neonate, pneumothorax, respiratory distress syndrome

## Abstract

Pneumothorax is a rare cause of respiratory distress in the newborn. We report our experience with a full-term female neonate who had primary spontaneous pneumothorax. No risk factors were identified. The pneumothorax was resolved completely with conservative management.

## Introduction

Pneumothorax is the presence of air between the parietal and visceral pleura of the thoracic cavity, with a consequent increase in intrapulmonary pressure. Like the Hermansen study [1], Lorah et al. found the incidence of spontaneous pneumothorax is 1-2% in term neonates, but it increases to about 6% in premature neonates. Pneumothorax can be secondary to infection, meconium aspiration, or ventilation barotrauma. The identified risk factors that can contribute to neonatal pneumothorax are male gender, low birth weight, prematurity, post-maturity, and aggressive resuscitation at birth [2]. Usually, affected neonates will develop acute respiratory distress soon after birth. Early suspicion and diagnosis are important. Management rarely exceeds conservative measures [2].

## Case presentation

**Figure 1:**
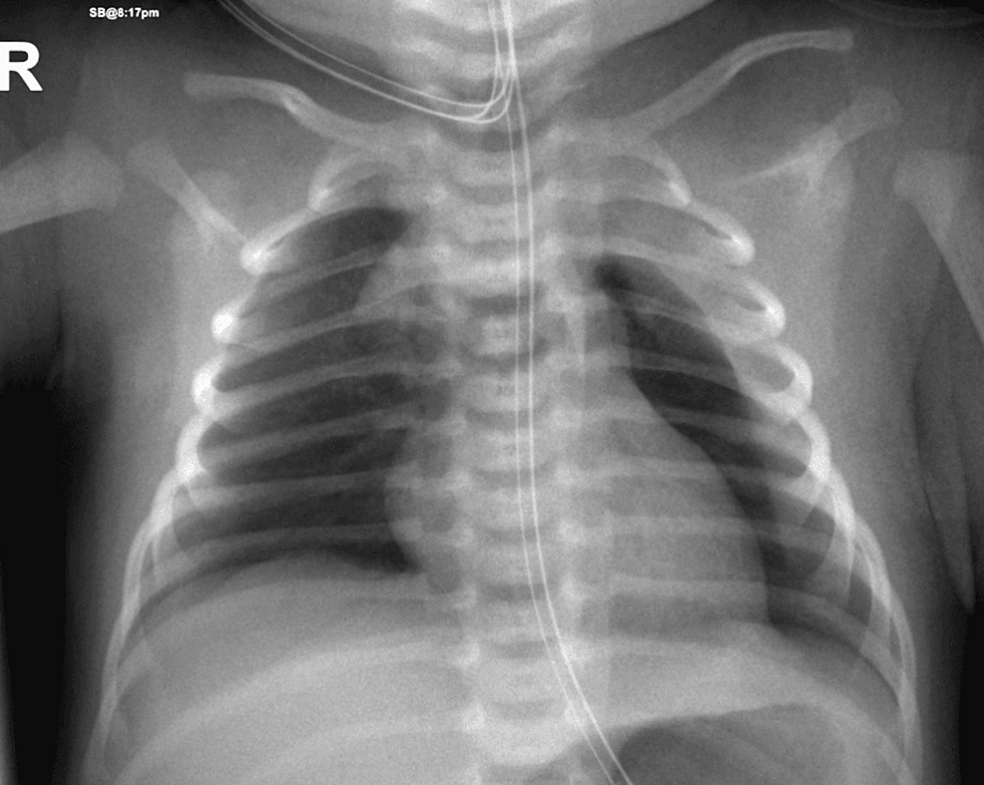
Left-sided pneumothorax Left-sided pneumothorax associated with a moderate amount of left-sided pleural effusion with a complete collapse of the left lung

A full-term female neonate was delivered via spontaneous vaginal delivery with unremarkable antenatal history. There was no history of prolonged rupture of membrane or meconium-stained liquor, and she cried immediately. Apgar scores at birth were 8 and 9 at the first and fifth minutes, respectively. She started to have cyanosis and grunting at 10 minutes of life. On examination, she was a healthy baby with no dysmorphic features. Her birth weight was 2730g. The respiratory rate was 60 cycles per minute and the heart rate was 160 beats per minute. There was respiratory distress with nasal flaring and mild subcostal recession. Breath sounds were decreased bilaterally without any crepitations or wheezing. Other systemic examinations were unremarkable. Non-invasive ventilation (continuous positive airway pressure (CPAP): positive end-expiratory pressure (PEEP) 5, FiO2 30%) was initiated, and venous blood gas was done in Table 1. The first chest X-ray is shown in was obtained on the first day of life, which showed left-sided pneumothorax (Figure 1).

**Table 1:** Venous blood gas VBG - venous blood gas

Variables	pH	pCo2 (mmHg)	HCO3 (mmol/L)	BE	Lactate
VBG at birth	7.28 (7.31 - 7.41)	53 (41 - 51)	16 (23 - 28)	-7.9 (-2 - 3)	6.6
VBG after two hours	7.34 (7.31 - 7.41)	42 (41 - 51)	22 (23 - 28)	-3 (-2 - 3)	3.3

**Figure 2:**
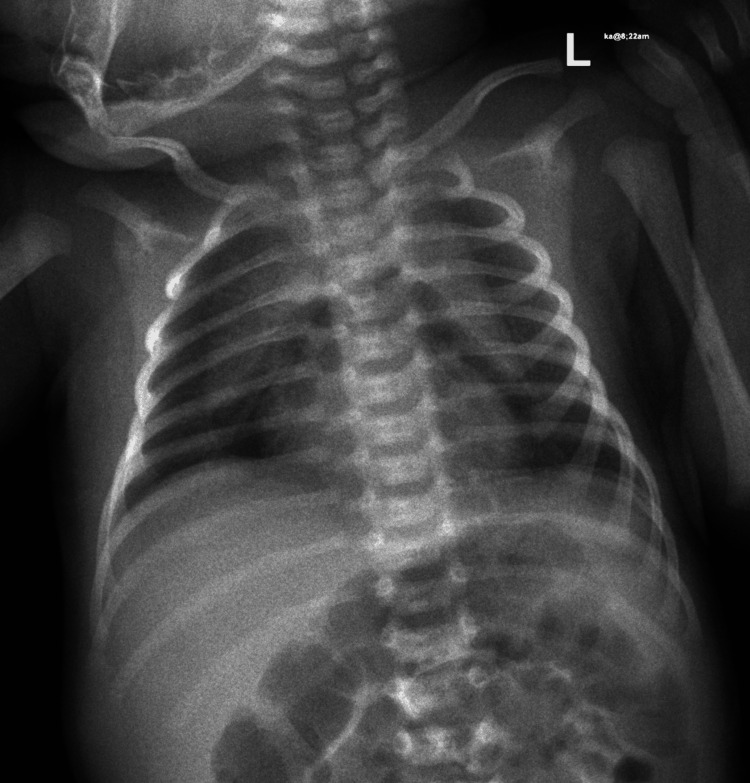
Pneumomediastinum Showed evidence of pneumomediastinum (air below the thymus)

Echocardiography was normal with no signs of pneumopericardium. The sepsis screen was negative. CPAP was applied for around nine hours then as her respiratory distress was improving, she was weaned to low flow oxygen 0.1-1 liters per minute via nasal cannula for 24 hours, then weaned to room air. Repeated chest X-ray after six days (Figure 2) showed evidence of pneumomediastinum.

On day nine, the CT chest showed mild-to-moderate anterior pneumomediastinum inferior to the thymus.

**Figure 3:**
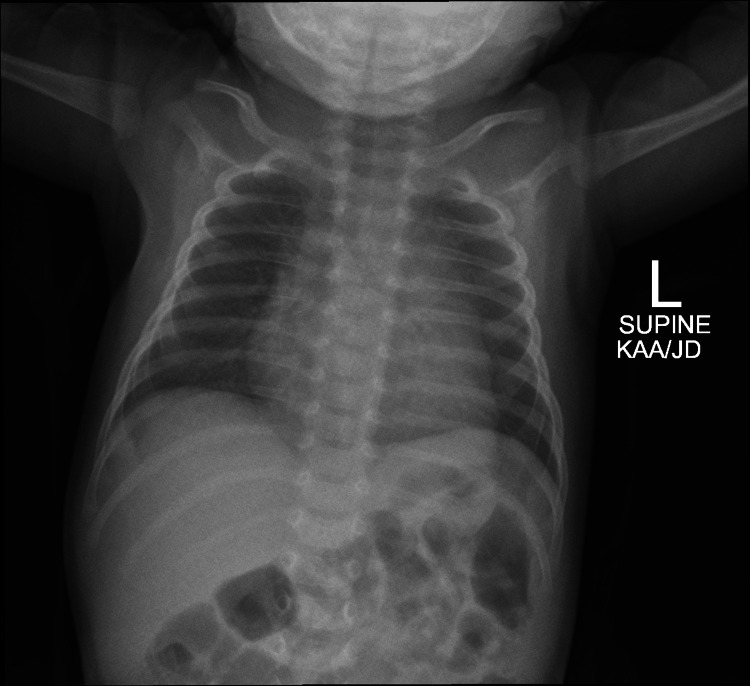
Normal chest X-ray

She was discharged home on day nine because she remained stable with no signs of respiratory distress. She did not require any surgical intervention in the form of chest tube placement or needle thoracentesis. After one month she was seen in the pulmonology clinic. She was active with no respiratory distress. Her O2 saturation was 99% on room air. She was thriving well; her weight was 3.9 kg (Z-score -0.67). Chest exam revealed equal vesicular breath sound bilaterally with no added sound. Repeated chest X-ray was normal (Figure 3). 

## Discussion

Respiratory distress is common in the early neonatal period. Multiple conditions can cause respiratory distress in term infants. The most common causes are Transient tachypnoea of the newborn, meconium aspiration syndrome, cardiac failure, pneumonia, and pneumothorax. Pneumothorax can be spontaneous or secondary to an underlying lung disease such as pneumonia, meconium aspiration, or mechanical ventilation [2].

Currently, there are no guidelines for effectively managing spontaneous pneumothorax in full-term neonates. The management of this condition can vary depending on the severity of the case. In less severe cases, careful observation may be employed, while more severe cases may require intercostal drainage to relieve the respiratory distress. Early detection is crucial, and aspiration of persistent pneumothoraxes in symptomatic infants should be considered [2]. Diagnosis can be delayed, especially if not suspected. In one study, it was shown that it takes between 0.5 and 27 hours before the clinical diagnosis is made. In that study, 46% of the neonates with pneumothorax displayed specific radiological signs such as the double diaphragm sign, enhanced cardio-mediastinal sharpness, deep sulcus sign, and basilar hyperlucency. These radiological signs can be helpful for early diagnosis [3].

Most cases won't require intervention, but chest tube insertion should be considered to allow lung expansion in symptomatic infants. Only 7.5% of symptomatic term infants with spontaneous pneumothorax would require chest tube insertion or thoracentesis [4]. The chest tube can be clamped for at least 24 hours after cessation of air leak. If there is no recurrence of pneumothorax, the chest tube can be removed [5]. In full-term or near-term neonates with spontaneous pneumothorax, the overall outcome is generally good. Infants who require prolonged oxygen therapy or positive pressure ventilation will have higher morbidity. In rare cases, subcutaneous emphysema and pulmonary hemorrhages may occur. Late complications such as bronchopulmonary dysplasia and neurodevelopmental impairment have also been documented. Neonates with primary lung disorders, such as congenital pneumonia or meconium aspiration syndrome at a high risk of developing pneumothorax and its associated complications [6]. It is crucial to closely monitor these cases and provide appropriate treatment and care to ensure the best possible outcomes. The goal is to optimize treatment approaches and minimize complications associated with this condition. Early detection and timely intervention are key factors in achieving better outcomes and reducing long-term morbidity [5,6].

The following table summarizes previously reported cases of term neonates with spontaneous pneumothorax without underlying conditions (Table 2).

**Table 2:** Comparing multiple case reports The following table summarizes previously reported cases of term neonates with spontaneous pneumothorax without underlying conditions

Author	Gestational age	Gender	Age at presentation	Presenting symptoms	Management	outcome
Adekoya et al. [7]	38 weeks	Male	At birth	Tachypnea with increased work of breathing	Oxygen	Resolved spontaneously
Gharibvand et al. [6]	Term	Male	At birth	Respiratory failure and shock	Intubated with no intervention	Extubated within two days and pneumothorax resolved spontaneously
Rocha et al. [8]	39 weeks	Male	Four hours of life	Grunting with subcutaneous emphysema	Oxygen	Resolved spontaneously at day nine
Karthikeyan et al. [9]	40 weeks	Male	At birth	Desaturation to 85% with increased work of breathing	Intubated with a chest tube. Antibiotic	Improved after 10 days
Haley et al. [10]	40 weeks	Male	At birth	Desaturation to 80% with tachypnea	Oxygen	Resolved spontaneously
Huseynov et al. [11]	36+5 weeks	Male	24 hrs	Respiratory distress	Chest tube with blood patch	Improved after 2 days
James et al. [12]	Term	Female	Within 1 hour of birth	Respiratory distress with desaturation 70%	Bilateral needle thoracentesis	Resolved completely

## Conclusions

Spontaneous pneumothorax in term is rare but a well-recognized cause of respiratory distress in the newborn. Timely diagnosis and adequate management reduce morbidity. Chest X-rays need to be considered in newborns with respiratory distress to rule out spontaneous pneumothorax because early recognition is lifesaving. Most cases resolve with conservative treatment and intervention is rarely required. Close and direct observation is the key to monitoring any respiratory deterioration. Spontaneous pneumothorax might require chest tube insertion if it causes severe respiratory distress or hemodynamic instability, which was reported in some cases.
